# Psychosocial risks among the healthcare workforce working in COVID services: findings from a cross-sectional study on psychosocial risks

**DOI:** 10.1192/j.eurpsy.2022.653

**Published:** 2022-09-01

**Authors:** P.A. Gouveia, D. Lopes, A.R. Henriques, P. Moura, A. Pires

**Affiliations:** 1 Local health Unit of Lower Alentejo, Psychiatry, Beja, Portugal; 2 Comprehensive Health Research Centre; & EpiDoC Unit, CEDOC, Nova Medical School, Universidade Nova De Lisboa, LISBOA, Portugal

**Keywords:** covid, ocupational psychiatry, healthcare workers, psychosocial risks

## Abstract

**Introduction:**

Poor management in healthcare can have significant consequences in the workers’ health, performance, and quality of care. Several risks worsened during the COVID-19 pandemic, namely among the workforce caring for patients with suspected/confirmed COVID-19 infection.

**Objectives:**

We aimed to assess psychosocial risks among a sample of 235 healthcare workers deployed in COVID-19-related services in Portugal’s Lower Alentejo.

**Methods:**

Participants filled out with ten sociodemographic questions and the Euro-Portuguese medium version of the COPSOQ II questionnaire. Data collection occurred February 2021. Tertiles were used to render a traffic light risk categorization. Results were processed with qualitative and quantitative descriptive statistical analysis. To compare groups relative to each outcome, t-tests were used for variables with two categories. Whenever data was not normally distributed, Mann-Whitney tests were used. For variables with more than two groups non-parametric Kruskal-Wallis was applied. Bonferroni correction was also applied, testing each individual hypothesis at the level of significance of α_i_=0.05/29. A statistically significant difference between two groups did not necessarily yield a different risk colour.

**Results:**

Overall, cognitive demands, emotional demands and influence at work showed the highest risk, while 19 domains showed intermediate risk. The burnout domain showed to be highest among nurses and operational assistants working in the Intensive Care Unit. Several associations between COPSOQ domains and sociodemographic variables are also discussed.

**Conclusions:**

Assessment of psychosocial stressors in healthcare units is needed to promote risk reduction policies and workplace reforms. Accessible occupational services, therapeutic and rehabilitative strategies should play a role in improving health hazards in unhealthy workplaces.

**Disclosure:**

No significant relationships.

